# Conformational transitions regulate the exposure of a DNA-binding domain in the RuvBL1–RuvBL2 complex

**DOI:** 10.1093/nar/gks871

**Published:** 2012-09-21

**Authors:** Andrés López-Perrote, Hugo Muñoz-Hernández, David Gil, Oscar Llorca

**Affiliations:** ^1^Centro de Investigaciones Biológicas (CIB), Consejo Superior de Investigaciones Científicas (CSIC), Ramiro de Maetzu 9, 28040 Madrid and ^2^Structural Biology Unit, CIC bioGUNE, Parque Tecnológico de Bizkaia, 48160 Derio, Bizkaia, Spain

## Abstract

RuvBL1 and RuvBL2, also known as Pontin and Reptin, are AAA+ proteins essential in small nucleolar ribonucloprotein biogenesis, chromatin remodelling, nonsense-mediated messenger RNA decay and telomerase assembly, among other functions. They are homologous to prokaryotic RuvB, forming single- and double-hexameric rings; however, a DNA binding domain II (DII) is inserted within the AAA+ core. Despite their biological significance, questions remain regarding their structure. Here, we report cryo-electron microscopy structures of human double-ring RuvBL1–RuvBL2 complexes at ∼15 Å resolution. Significantly, we resolve two coexisting conformations, compact and stretched, by image classification techniques. Movements in DII domains drive these conformational transitions, extending the complex and regulating the exposure of DNA binding regions. DII domains connect with the AAA+ core and bind nucleic acids, suggesting that these conformational changes could impact the regulation of RuvBL1–RuvBL2 containing complexes. These findings resolve some of the controversies in the structure of RuvBL1–RuvBL2 by revealing a mechanism that extends the complex by adjustments in DII.

## INTRODUCTION

RuvB-like 1 (RuvBL1), also known as Rvb1, Pontin, TIP49 and TIP49A, and RuvB-like 2 (RuvBL2), also known as Rvb2, Reptin, TIP48 and TIP49B, are highly conserved ATPases that belong to the AAA+ (ATPases associated with diverse cellular activities) family (1). RuvBL1 and RuvBL2 share 65% of sequence similarity, and they are homologous to prokaryotic RuvB, a protein that together with RuvA and RuvC provides the energy for the resolution of Holliday junctions, a DNA intermediate in many DNA repair processes (2). RuvBL1 and RuvBL2 are essential components of several unrelated multi-protein complexes (3), including INO80 and SWR1 chromatin remodelling complexes (4), the TIP60 histone acetyltransferese complex (5), the R2TP complex involved in biogenesis of small nucleolar ribonucleoproteins (snoRNPs) (6,7) and complexes that regulate the activity of phosphatidylinositol 3-kinase (PI3K)-like kinases (8). RuvBL1 and RuvBL2 have been implicated in multiple and essential functions in the cell (1,3), including transcription (9), DNA repair (8), nonsense-mediated mRNA decay (NMD) (8) and telomerase assembly (10). In addition, several studies have described a link between deregulation of RuvBL1 and RuvBL2 and some types of cancer (11–13).

The function of RuvBL1 and RuvBL2 in the context of such diverse sets of complexes is unclear, but current models propose that they act as scaffolds for multi-protein interactions and that their ATPase activity could be important for regulatory steps performed during chromatin remodelling and telomerase assembly (1,3). For instance, human RuvBL1 and RuvBL2 interact with components of telomerase, contributing to the biogenesis of a functional enzyme that requires the ATPase activity of RuvBL1 (10). In yeast, RuvBL1 and RuvBL2 homologues recruit Arp5 to assemble a catalytically active INO80 remodelling complex (14). RuvBL1 and RuvBL2 are also involved in the assembly of complexes containing PI3K-related protein kinases (PIKKs), such as ATM, ATR, mTOR and SMG-1 (15). RuvBL1 and RuvBL2 regulate the functions of SMG-1 and contribute to NMD in mammals (8).

**Figure 1.**
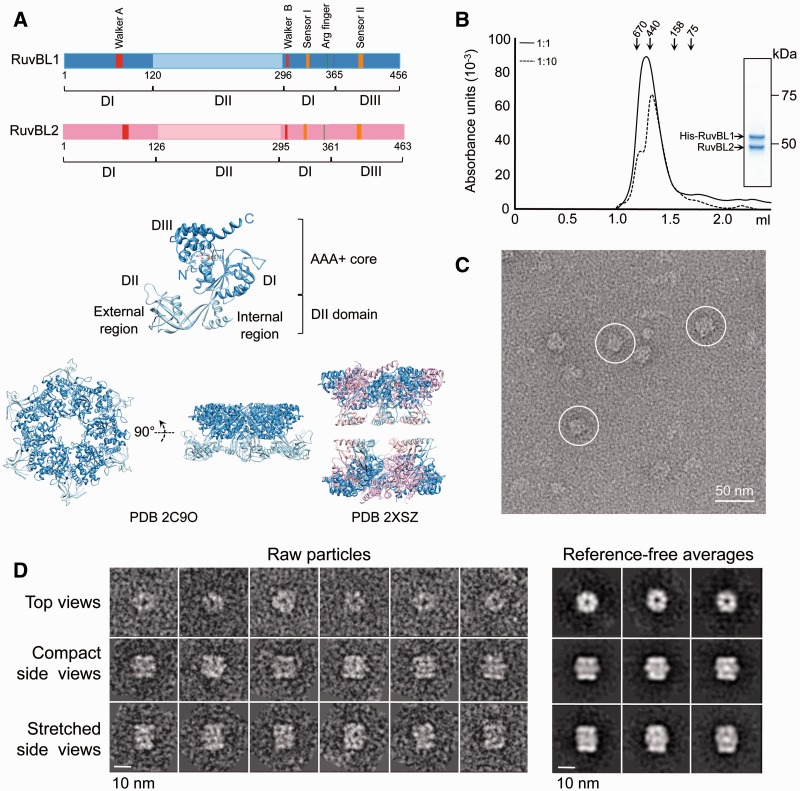
Purification and electron microscopy of human RuvBL1–RuvBL2. (**A**) Sequence of RuvBL1 and RuvBL2 and atomic structures of homo-hexameric RuvBL1 (PDB code: 2C9O) (16) and the truncated double-ring RuvBL1–RuvBL2 complex (PDB code: 2XSZ) (17). The middle panel shows an enlarged view of a RuvBL1 monomer. Domains and the N- and C-terminal ends of the protein are labelled. Colour codes for different domains in the primary structure are used in the atomic structures. (**B**) Elution profile from a SEC of the His–RuvBL1–RuvBL2 complex after affinity purification (solid line). The sample was also analysed after 1:10 dilution (dashed line). Molecular weight standards corresponding to 670, 440, 158 and 75 kDa are indicated. Inset shows a SimplyBlue (Novagen) stained SDS–PAGE of purified RuvBL1–RuvBL2 complex after SEC. (**C**) Representative field of an electron micrograph obtained for RuvBL1–RuvBL2 after negative staining. Selected side view images are highlighted. Scale bar, 50 nm. (**D**) Raw images and reference-free 2D averages obtained after reference-free classification and averaging. Scale bar, 10 nm.

Recently, X-ray crystallography and electron microscopy (EM) have provided important insights about the structure of RuvBL1 and RuvBL2 proteins from human and yeast. A high-resolution structure of human RuvBL1 showed that the protein assembles as a hexameric ring, similar to what has been described for other members of the AAA+ family, including RuvB (16) (Figure 1A). Each monomer consists of three distinct domains. Domain I (DI), residues 1–120 and 296–365, and domain III (DIII), residues 366–456, make up the AAA+ core of the protein. This core oligomerizes in the archetypical hexamer observed in many AAA+ proteins, and it contains the so-called Walker A and Walker B motifs responsible for the ATPase activity. Domain II (DII), a ∼170 amino acid insertion comprising residues 121–295, is connected to the core by a linker containing two β-strands that permit some flexibility. DII is spatially organized into the following two regions: internal and external. The internal region comprises two α-helices and loops, whereas interestingly, the external region resembles the DNA-binding domain of several proteins, including replication protein A (RPA) (16) (Figure 1A). Previous studies have shown that the RuvBL1 DII domain binds single-stranded DNA (ssDNA), double-stranded DNA (dsDNA) and single-stranded RNA (ssRNA) *in vitro* (16), whereas some data also suggest a potential role in protein binding (18). Evidence for nucleic acid binding to the DII domain from RuvBL2 has not yet been provided.

Several reports have described that human RuvBL1 and RuvBL2 can assemble a dodecameric complex containing two hexameric rings. A recent crystal structure of RuvBL1–RuvBL2, in which most of DII was truncated (lacking RuvBL1 residues 127–233 and RuvBL2 residues 134–237), revealed that these proteins formed a dodecamer consisting of two heterohexameric rings with alternating subunits in each ring (17). In the structure, the two hexameric rings are bound back-to-back, and the interaction between rings is mediated, in part, by segments in the internal region of DII still present in these constructs. Interestingly, each RuvBL1 subunit in one of the rings interacts with a RuvBL2 subunit in the other ring. Surprisingly, a negative stain EM reconstruction of the human dodecameric complex differs dramatically from the crystallographic structures (16,17,19). To explain such a discrepancy, it has been argued that RuvBL1 and RuvBL2 could potentially assemble several types of complexes to accommodate the diversity of its functions *in vivo* (20,21).

These findings for the human proteins have been complemented by EM studies in yeast. Yeast homologues of RuvBL1 and RuvBL2 have been described as heterohexameric single rings (22). The yeast proteins also assemble double-ring complexes, with DII domains facing the interface between rings (23,24). Interestingly, two independent structures of yeast double-ring complexes are significantly different. The cryo-EM structure by Torreira *et al.* (24) is compact, and DII domains are interconnected, whereas the structure by Cheung *et al.* (23) is elongated, and the distance between the two rings is significantly larger.

It has been proposed that yeast homologues of RuvBL1 and RuvBL2 assemble *in vivo* as single hexameric rings only, whereas double-ring complexes would be artifacts induced by histidine tags in the recombinant proteins (22,23). However, human proteins have been found to assemble double rings under several experimental conditions (17,19,21). The interaction between RuvBL1 and RuvBL2 *in vitro* is affected by experimental conditions, such as the expression and purification of recombinant proteins. Therefore, it is difficult to assess the extent in which complexes identified *in vitro* will represent a faithful account of the oligomeric state *in vivo*. The relevant oligomeric states of RuvBL1 and RuvBL2 may also be influenced by their context in larger macromolecular complexes. It is conceivable that single- and double-ring complexes might have a functional significance.

A more complete structural understanding of RuvBL1 and RuvBL2 will provide an important platform for interpreting the assemblies they form while performing their diverse cellular functions. Although previous structural studies reveal that RuvBL1 and RuvBL2 form double-ring complexes, structures reported are different (17,19). Discrepancies have been interpreted as a consequence of the different experimental methods used for the production of complexes and/or potential inherent structural heterogeneity (17,20,23,24). A recent study using analytical centrifugation with human RuvBL1–RuvBL2 complexes raised the possibility that these proteins could be assembling a number of different complexes, at least *in vitro* (21). Here, we clarify many of these issues by resolving the structure of the full-length human RuvBL1–RuvBL2 dodecameric complex using cryo-EM of frozen hydrated samples, by improving the resolution of the structures compared with those published and by taking into account conformational heterogeneity using image-processing strategies. We describe a cryo-EM structure of the human RuvBL1–RuvBL2 dodecamer that is consistent with known crystal structures for parts of the complex. Moreover, we describe two conformations of the double-ring complex that coexist in the same preparation. These conformational changes impact in the exposure of putative DNA-binding regions in DII domains and could have a general role in regulating RuvBL1–RuvBL2. We define the structural basis for this conformational heterogeneity and propose a reasonable explanation for the differences observed in previously reported structures.

## MATERIALS AND METHODS

### Expression and purification of human RuvBL1–RuvBL2 complexes

N-terminal His_10_-tagged human RuvBL1 was cloned using a modified pET15b (Novagen) vector, pETEV15b, which includes 10-histidines residues and a tobacco etch virus (TEV) protease cleavage site (kindly provided by Dr J.M. Pereda, CIC Salamanca, Spain). Untagged human RuvBL2 was cloned using a pCDFDuet-1 (Novagen) vector. Both constructs were co-transformed into *Escherichia coli* BL21 (DE3) cells grown in LB medium. Overexpression was induced at 28°C for 3 h by addition of 0.1 mM (final concentration) isopropyl-β-D-1-thiogalactopyranoside. Cell lysis was performed by sonication in lysis buffer (50 mM Tris–HCl pH 7.4, 500 mM NaCl, 10% (v/v) glycerol, 0.1% (v/v) NP-40) containing a cocktail of ethylenediaminetetraacetic acid -free proteases inhibitors (Roche). Lysate was cleared by centrifugation at 37 000 r.p.m. for 45 min at 4°C, and the supernatant was applied to a HisTrap HP column (GE Healthcare) equilibrated in buffer A (50 mM Tris–HCl pH 7.4, 300 mM NaCl, 10% (v/v) glycerol, 20 mM imidazole). Elution was performed using a 20–500 mM imidazole gradient, and fractions containing the His–RuvBL1–RuvBL2 complex were pooled and dialyzed overnight in buffer B (50 mM Tris–HCl pH 7.4, 250 mM NaCl). The sample was then applied to a size-exclusion chromatography (SEC) using a Superdex 200 PC 3.2/30 column (GE Healthcare) equilibrated in buffer B. Final protein concentration was estimated by measuring of absorbance at 280 nm. Purification was monitored by sodium dodecyl sulphate-polyacrylamide gel electrophoresis (SDS–PAGE) and SimplyBlue (Novagen) staining.

For those experiments where the His-tag of His–RuvBL1 was removed, fractions pooled from the HisTrap HP column (GE Healthcare) were incubated overnight with TEV protease at 4°C while dialyzing against buffer A. The sample was reapplied to the HisTrap HP column and eluted as described earlier in the text to separate the untagged complex (flow-through) from any residual His–RuvBL1–RuvBL2 complex, the His-tag and the TEV protease (which includes a N-terminal His_6_-tag). Flow-through from the affinity column was applied to a Superdex 200 PC 3.2/30 column (GE Healthcare) and fractionated under the same conditions as those described for the tagged version of the complex (see earlier in the text). Purification of the untagged RuvBL1–RuvBL2 complex was monitored by SDS–PAGE and SimplyBlue (Novagen) staining and western blot with an anti-His antibody (monoclonal anti-polyhistidine peroxidase conjugate, Sigma-Aldrich).

### Blue native PAGE

Fifteen microlitres of purified His–RuvBL1–RuvBL2 or RuvBL1–RuvBL2 complexes (both at 1 mg/ml) was mixed with 4 µl of loading dye and was loaded into blue native PAGE (BN–PAGE) gels (NativePAGE Novex Bis-Tris Gel System, Novex). Electrophoresis was performed following manufacture’s instructions. The presence of the tag was monitored by western blot with an anti-His antibody (monoclonal anti-polyhistidine peroxidase conjugate, Sigma-Aldrich).

### EM and image processing of negative stained images

A few microliters of freshly purified complexes (0.01 mg/ml) were deposited on carbon-coated grids and were stained using 2% (w/v) uranyl formate. Micrographs were recorded using a JEOL 1230 transmission electron microscope and a 4 k × 4 k TVIPS CMOS detector using a low-dose protocol under control of the EM-TOOLS software (TVIPS). Images were collected at a final magnification of 68 222×, and these were down-sampled to a final 4.56 Å per pixel. The contrast transfer function (CTF) for each micrograph was estimated using CTFFIND3 (25), and correction was performed using BSOFT (26). Ten thousand particles were extracted from the micrographs. Particles were classified and averaged in 2D using the *e2refine2d.py* command in EMAN2 (27). Roughly, 6000 side views from the data set were split, and these were used for angular refinement using EMAN (28). Templates for starting angular refinement were obtained by applying the *startcsym* command in EMAN (28) to side and top view averages. Given the high degree of sequence similarity between RuvBL1 and RuvBL2 (16), the two proteins cannot be distinguished at the resolution provided by negative stain experiments; thus, 6-fold rotational symmetry (C6 symmetry) was assumed through refinement. In addition, the crystal structure of the truncated RuvBL1–RuvBL2 dodecamer revealed the symmetry between the two rings (dihedral rotational symmetry, D2) (17), which was assumed throughout refinement. The resolution of the compact and stretched conformations was estimated as 26 Å and 30 Å, respectively, using Fourier shell correlation (FSC) with a 0.5 cut-off.

In an independent set of experiments, *ab initio* structures of the RuvBL1–RuvBL2 complexes were obtained using the random conical tilt (RCT) method (29) as implemented in EMAN2 (27). A total of 3200 tilt pairs of images were collected and classified into 30 averages, each with ∼100 particles. Representative averages were selected to obtain their 3D structure, imposing C6 + D2 symmetry. All structures were displayed using UCSF Chimera (30).

### EM of His–RuvBL1–RuvBL2 incubated with nucleotides

Fractions from the SEC enriched in His–RuvBL1–RuvBL2 dodecamers (protein concentration = 0.01 mg/ml) were pooled and then split for four independent experiments. One of the experiments was incubated in buffer B without adding any nucleotide as a control sample. In independent experiments, the sample was incubated in buffer B supplemented with 2 mM ADP and 2 mM MgCl_2_ or two non-hydrolyzable nucleotide-metal fluoride analogues with 5 mM MgCl_2_ for 1 h at 30°C. Nucleotide analogues were prepared by pre-incubation of 1 mM ADP with 30 mM NaF and 5 mM BeCl_3_ (ADP–BeFx, pre-hydrolytic state) or 5 mM AlCl_3_ (ADP–AlFx, transition state) at 30°C for 10 min*.* Each reaction was applied on glow-discharged carbon-coated grids and was stained using 2% (w/v) uranyl formate. Grids were imaged as described for other negative stain samples (see earlier in the text), and micrographs were recorded on a 4 k × 4 k TVIPS CMOS detector. Particles were extracted using EMAN2 (27). Classification and averaging in 2D were performed using the *e2refine2d.py* command in EMAN2 and the *cl2d* command in XMIPP (31), obtaining similar results. Cl2d is a 2D multi-reference alignment and classification procedure based on the hierarchical clustering approach (32). The number of particles assigned to each class was quantified.

### Cryo-EM and 3D reconstruction

The purified His–RuvBL1–RuvBL2 complex was applied to QUANTIFOIL® 300 mesh R2/1 holey carbon copper grids (Quantifoil® Micro Tools GmbH, Jena, Germany) and vitrified using a Vitrobot Mark III (FEI Inc., Eindhoven, The Netherlands). Vitrified samples were analysed in a JEM-2200FS FEG electron microscope using an in-column energy filter (Omega Filter) and a 626 cryo-holder (Gatan Inc., Warrendale, PA, USA). A total of 338 images were collected on a 4 k × 4 k UltraScan 4000 CCD camera (Gatan Inc., Pleasanton, CA, USA) at a final magnification of 86 855.8×, 1.73 Å per pixel. Micrographs were recorded using a defocus range of 1.2–3.5 micron underfocus. Micrographs were pre-processed using XMIPP (31) and binned to 3.45 Å per pixel before CTF correction using CTFFIND3 (25) and BSOFT (26). A total of 24 500 particles were boxed using XMIPP (31), and the particles were subsequently classified and averaged in 2D using a 2D multi-reference alignment and classification based on the hierarchical clustering approach described earlier in the text (*cl2d* command) (31,32). This classification procedure enabled splitting images that corresponded to the two conformations of the complex. Each data set was processed independently using only side views. Angular refinement was performed using the *refine* command in EMAN and using the *startcsym* command to generate the initial templates (28). Further refinement was performed using the *projection_matching* command in XMIPP (31). C6 and D2 rotational symmetry was assumed throughout refinement, as the potential differences in structure between RuvBL1 and RuvBL2 are beyond the resolution limits in this work. After convergence, the symmetry was relaxed to 3-fold rotational symmetry and the structure remained unchanged confirming that any potential difference in structure between RuvBL1 and RuvBL2 in the complex is beyond the resolution obtained in this work. The handedness of the reconstructions was determined by comparison with the crystal structure of the truncated RuvBL1–RuvBL1 complex (17). This crystal structure defined the interaction between the top and bottom rings through RuvBL1 and RuvBL2 subunits from opposite rings. These interactions were only preserved in one of the two possible hands for the cryo-EM reconstruction. A maximum likelihood approach (33) and a multi-reference refinement strategy using the *multi-refine* command in EMAN (28) were used to evaluate the presence of multiple conformers in the data set. The resolution was estimated as 15 Å and 16 Å for the compact and stretched conformations, respectively, by FSC (cut-off of 0.5 correlation coefficient). The structures were displayed using UCSF Chimera (30).

### Pseudo-atomic modelling

Fitting of atomic structures for the human RuvBL1 hexameric ring (PDB code: 2C9O) (16) and the truncated human RuvBL1–RuvBL2 complex (PDB code: 2XSZ) (17) into the cryo-EM density were performed using UCSF Chimera (30). For the fitting of the DII domains into their cryo-EM density within the complex, the crystal structure of DII was extracted from the PDB (PDB code: 2C9O) (16), split in external and internal regions, and the fitting was performed by a combination of manual fitting and refinement using UCSF Chimera (30). Several constraints were applied during the refinement. For instance, connectivity between external and internal regions was preserved, and deviations from the crystal structure were minimized.

## RESULTS

### Human RuvBL1–RuvBL2 assembles as single and double-hexameric rings

Human His-tagged RuvBL1 (RuvBL1 hereafter unless specifically stated) and untagged RuvBL2 were cloned and co-expressed in *E. coli* cells to promote the assembly of the complex *in vivo*. RuvBL2 was found to bind to RuvBL1 in a 1:1 ratio after affinity purification of the complex. The interaction between RuvBL1 and RuvBL2 was maintained after SEC (input protein concentration in SEC ≈ 1 mg/ml) (Figure 1B). A calibration using molecular weight standards confirmed that we could resolve single- (∼440 kDa) from double-ring (∼670 kDa) oligomers under our experimental conditions. The SEC profile, covering the calibrated range, suggested a mixture of single- and double-ring complexes. Double-ring complexes were susceptible to disassembling at lower concentrations, as the same preparation was enriched in single rings after a 1:10 dilution (input protein concentration in SEC ≈ 0.1 mg/ml) (Figure 1B). Overall, these experiments suggest that single- and double-ring populations exist in a concentration dependent equilibrium. We found similar effects when the dilution experiment was performed after incubation with ADP or ATP (data not shown).

Fractions eluting from SEC in the region calibrated as double-ring complexes were observed in the electron microscope after negative staining, and the images obtained were compatible with a double-ring oligomer (Figure 1C). Roughly, 10 000 images were extracted from the micrographs, and these were classified and averaged using reference-free methods. Top views were abundant (40%) and displayed a clear ring shape as described previously for RuvBL1 and RuvBL2 (20) (Figure 1D). We also found rectangular views containing two intense bands of density at each end and a region of lower density at the centre. Based on the existing information for RuvBL1 and RuvBL2 (20) and the EM analysis of other ring-shaped complexes, these views were interpreted as side views of a double-ring oligomer. Intense bands at each end of the molecule could be assigned to the core ring of RuvBL1–RuvBL2, whereas the fainter densities at the centre corresponded to the DII domains (Figure 1D). Interestingly, we found two types of side views with either a short (37% of the images) or a long distance (23% of the images) between the putative AAA+ core rings, which we named ‘compact’ and ‘stretched’ conformations, respectively. Side views for hexameric single-ring complexes were not detected; thus, ring-shaped top views are likely enriched in single-ring complexes.

**Figure 2.**
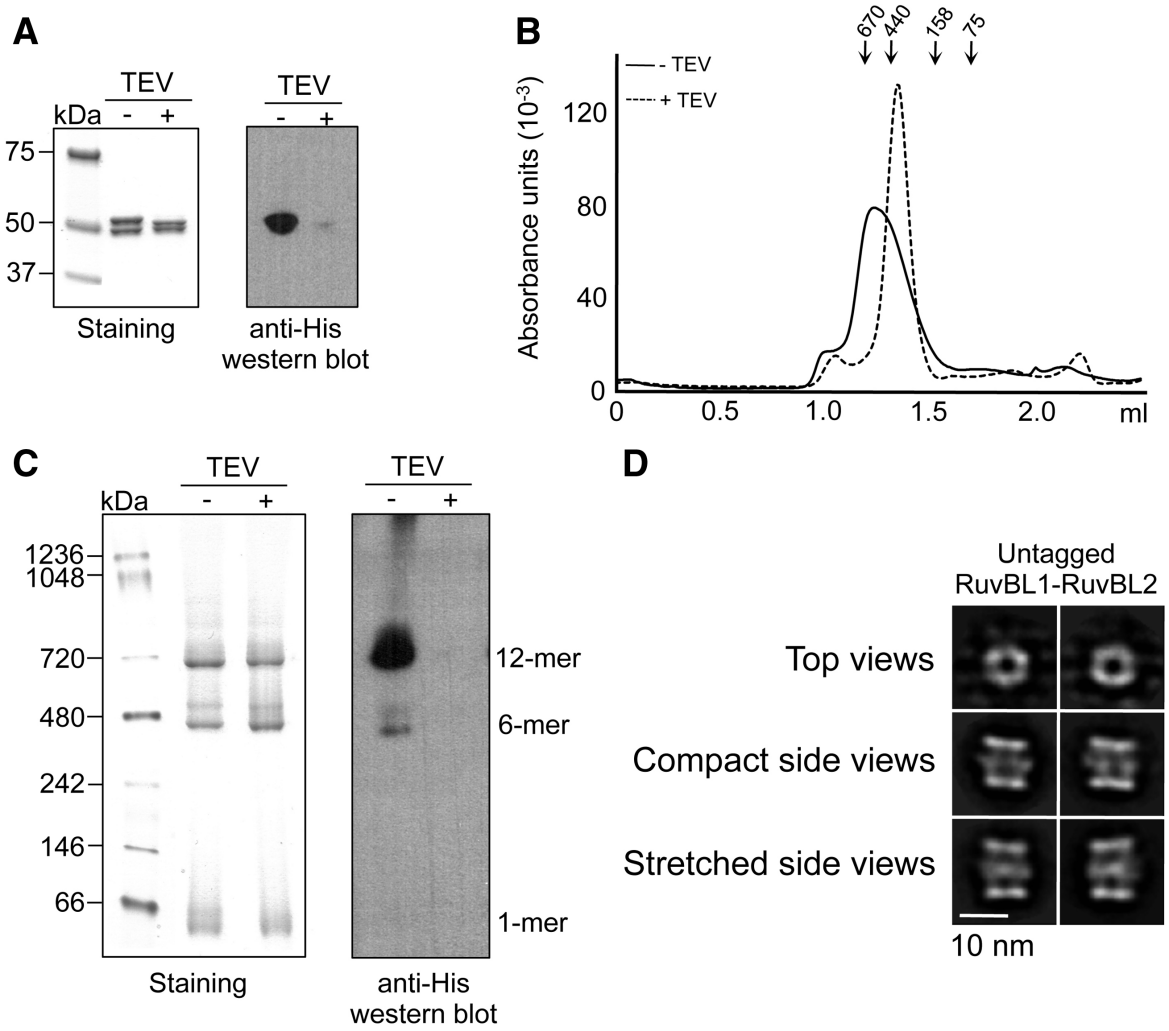
RuvBL1 and RuvBL2 assemble as a dodecameric complex in the absence of tags. (**A**) SDS–PAGE of purified untagged RuvBL1–RuvBL2 complex evaluated by Coomassie staining (left) and western blot against histidine tag (right). Addition of TEV (TEV+) removes the tag of the His-RuvBL1–RuvBL2 complex (TEV−). (**B**) SEC of His–RuvBL1–RuvBL2 (solid line, TEV−) compared with untagged RuvBL1–RuvBL2 (dash line, TEV+). Molecular weight standards corresponding to 670, 440, 158 and 75 kDa are indicated. (**C**) BN–PAGE of His–RuvBL1–RuvBL2 (TEV−) compared with untagged RuvBL1–RuvBL2 (TEV+). The different oligomeric species are indicated. Detection of the histidine tag in BN–PAGE was performed by western blot. (**D**) EM of untagged RuvBL1–RuvBL2. Particles were collected, classified and averaged. 2D reference-free averages for the different views of the complex are shown. Compact and stretched conformations were observed. Scale bar, 10 nm.

We evaluated the influence of the tag used for affinity purification in the ratio between hexameric and dodecameric complexes. His–RuvBL1–RuvBL2 complexes were purified by affinity chromatography, and the His-tag of RuvBL1 was removed by incubation with His-tagged TEV protease. Residual His–RuvBL1–RuvBL2, cleaved His-tag, and TEV protease were removed by reinjection onto the HisTrap column. Removal of the tag was confirmed by SDS–PAGE, as His–RuvBL1 migrates slower than RuvBL1, and western blotting detecting the histidine tag (Figure 2A). Purified untagged RuvBL1–RuvBL2 complex was subsequently analysed by SEC (Figure 2B), BN–PAGE (Figure 2C) and EM (Figure 2D). SEC (input protein concentration in SEC ≈ 0.8 mg/ml) revealed the disassembly of most of the double-ring complexes after removal of the tag (Figure 2B). This was probably a combined effect of removal of the tag and dilution of the sample, as shown in Figure 1B. On the other hand, BN–PAGE showed that both RuvBL1–RuvBL2 preparations, His-tagged and untagged, were enriched in double-ring complexes, but also contained a mixture of monomers and single-rings (Figure 2C). The abundance of double-ring complexes was similar in the tagged and untagged complexes. Importantly, we corroborated that the double-ring complexes resolved by BN–PAGE corresponded to untagged complexes by detecting the histidine tag by western blot. For further confirmation, the untagged RuvBL1–RuvBL2 complex was observed in the electron microscope, and 18 500 particles were collected and classified. Double-ring complexes in the compact and stretched conformations were detected for the untagged RuvBL1–RuvBL2 dodecamers, each conformation in a similar percentage to the one observed for the His-tagged complex (Figure 2D). Taken as a whole, these experiments indicate that double-ring complexes do exist in the absence of an affinity tag; thus, these are one of the possible conformers of the human RuvBL1–RuvBL2 complexes.

### Two coexisting conformations for RuvBL1–RuvBL2 double-rings

**Figure 3.**
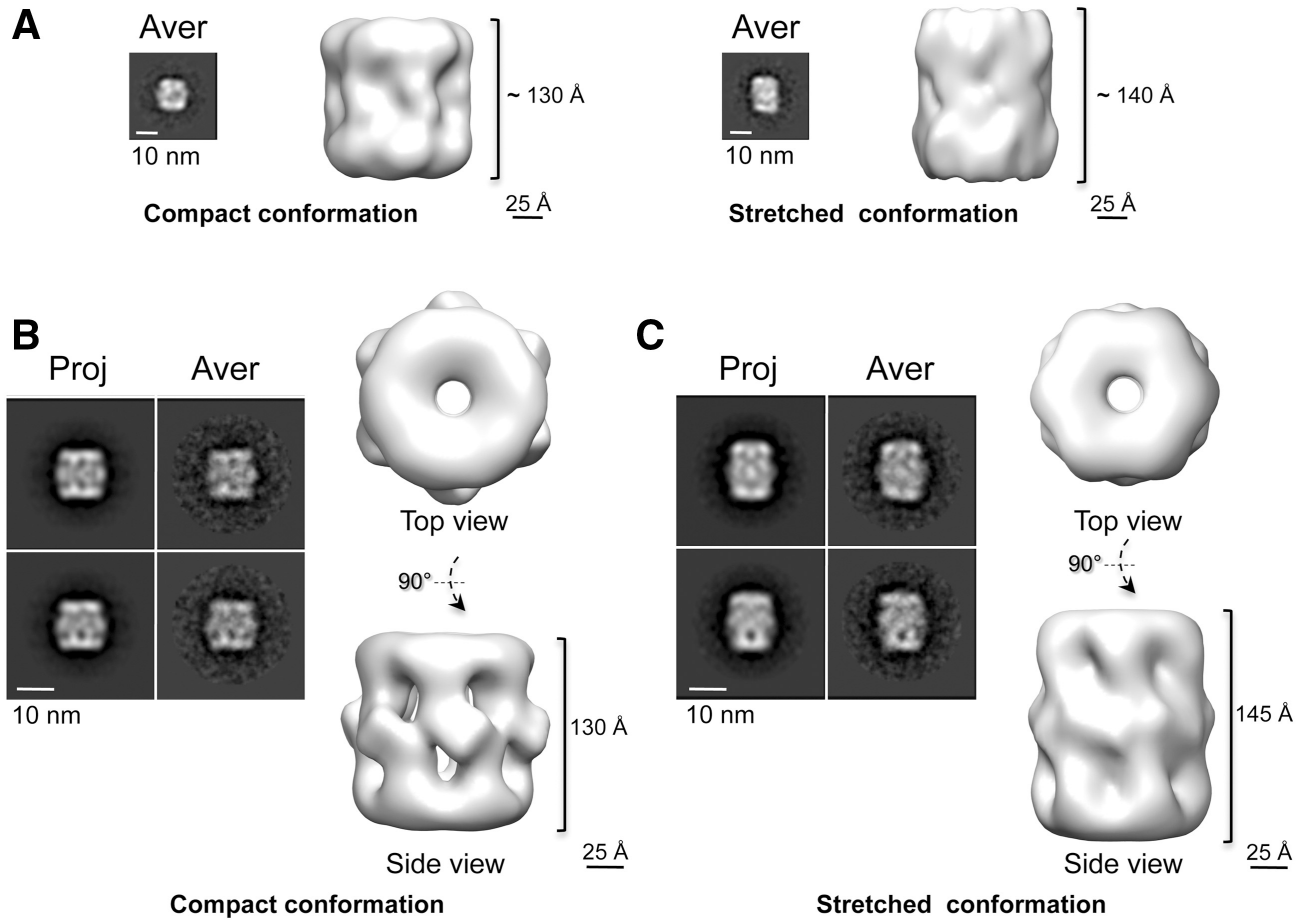
Two conformations of the human RuvBL1–RuvBL2 complex. (**A**) RCT structures reveal two coexisting conformations. Each panel shows the average (Aver) of those particles that were used for the RCT reconstruction together with one side view of the RCT structure. (**B**) Negatively stained structure of the compact conformation. Two representative pairs of projection (Proj) and average (Aver) after angular refinement are shown. (**C**) Negatively stained structure of the stretched conformation. Two representative pairs of projection (Proj) and average (Aver) after angular refinement are shown. Scale bar, 10 nm.

We obtained low-resolution *ab initio* structures of the RuvBL1–RuvBL2 complexes using the RCT method (29) (Figure 3A). This method exploits the relationship between tilted and untilted images of the same object to calculate a 3D structure from images corresponding to single views. The RCT structures for the short and long side view averages revealed that these images corresponded to distinct conformations of the complex with a significant difference in the length of the longitudinal axis (Figure 3A).

The resolution of the structures obtained from negatively stained images was improved by angular refinement methods using an independent data set (Figure 3B and C, Supplementary Figure S1A). After classification and averaging using reference-free methods, all further image processing was performed using only side views, as these are sufficient to reconstruct the complex. Particles corresponding to short (37% of the data set) and long (23% of the data set) side views were split in two groups and refined independently to obtain two structures, ‘compact’ (Figure 3B) and ‘stretched’ (Figure 3C). These two conformations corroborated the *ab initio* models obtained by the RCT approach applied to short and long side views. As a control, we compared ‘long’ and ‘short’ side view images to the ‘compact’ and ‘stretched’ conformations, respectively, which confirmed that the two types of views were incompatible, and they could not be derived from the same structure (data not shown).

The 26 Å resolution structure of the compact conformation obtained using negative staining revealed two rings at each end and six densities connected at the centre (Figure 3B). The EM structure was fitted with the atomic structure of RuvBL1 (16) (cross-correlation coefficient = 0.91) and the truncated RuvBL1–RuvBL2 complex (cross-correlation coefficient = 0.91) (17), revealing a strong resemblance between the X-ray and EM structures (Supplementary Figure S1B). The core AAA+ ring was placed at each end of the complex showing a central opening with similar dimensions to those found in the crystal structure of the RuvBL1 hexamer (16). The region at the centre, organized as six bulky densities projecting slightly outwards, could then be assigned to the DII domains missing in the crystallized construct (17). In contrast, the 30 Å resolution structure of the stretched complex displayed a different conformation (Figure 3C). The AAA+ rings could be fitted with crystal structure of the AAA+ core (cross-correlation coefficient = 0.92) (17) (Supplementary Figure S1B), but the centre of the molecule was elongated and the DII domains were noticeably extended. Clearly, the length of this conformation was incompatible with that of the RuvBL1–RuvBL2 dodecamer crystal structure (17), which could not be fitted satisfactorily within the EM density.

**Figure 4.**
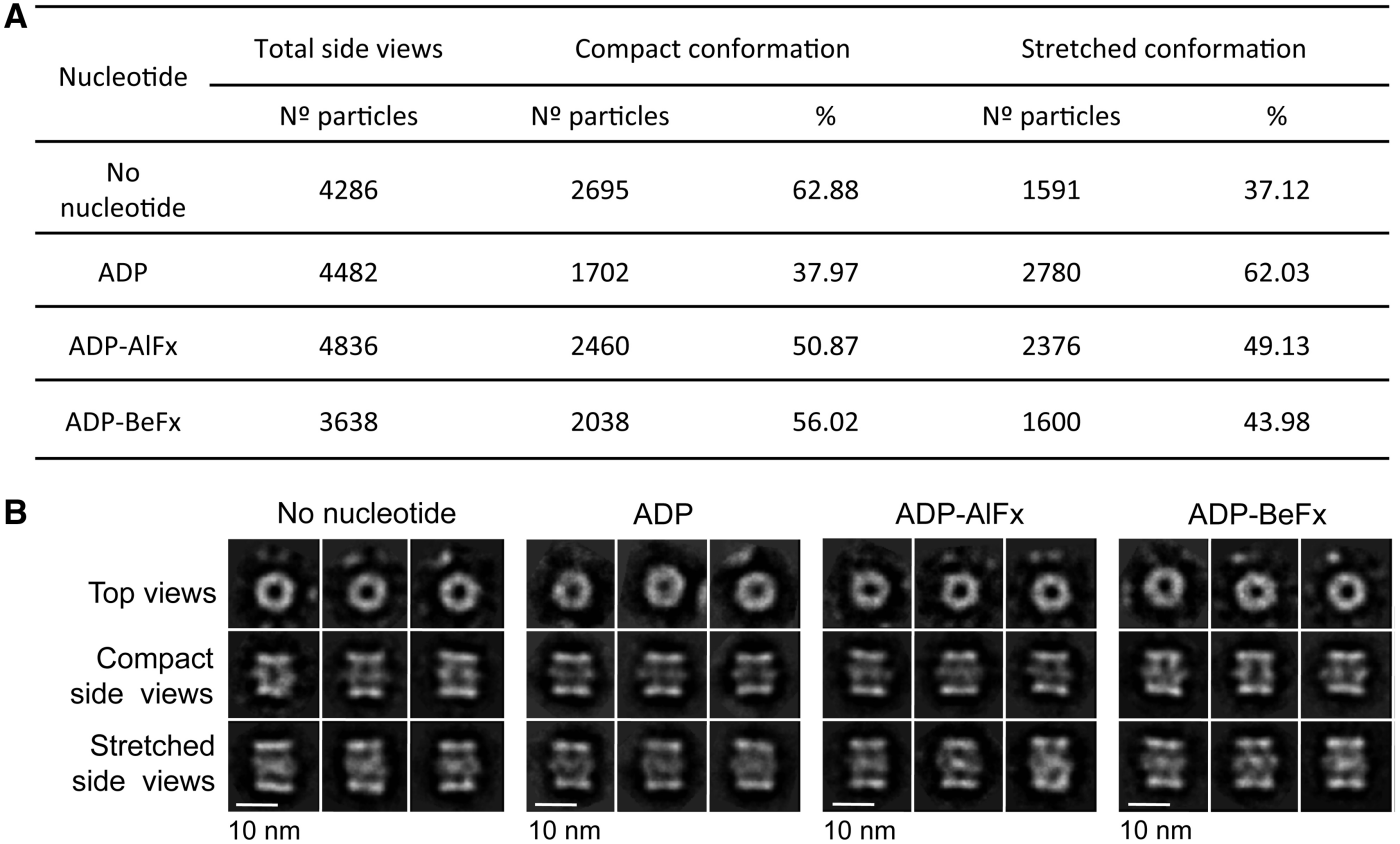
Influence of nucleotides in the ratio between compact and stretched conformations. (**A**) Percentage of side view particles classified as compact and stretched quantified after incubation with different nucleotides. (**B**) 2D averages obtained for each condition are also shown. Scale bar, 10 nm.

We tested whether the transition from the compact to the stretched conformation could be modulated by nucleotides. Fractions from a SEC enriched in RuvBL1–RuvBL2 dodecamers were pooled, and the resulting preparation was split for four independent experiments. The RuvBL1–RuvBL2 complex was incubated with ADP–Mg^2+^, the nucleotide analogue ADP–BeFx (pre-hydrolytic state) or the nucleotide analogue ADP–AlFx (transition state) (Figure 4). A control was also performed without adding any nucleotide. Each sample was analysed in the electron microscope, and images collected were processed and classified to determine the number of particles corresponding to top views and compact and stretched side views (Figure 4A). 2D averages for each experiment were also obtained (Figure 4B). All experiments were performed in duplicate, with identical results. Compact and stretched conformations co-existed in all conditions, with ADP showing a slight increase in the percentage of molecules found in the stretched conformation. We found that the percentage of top views was approximately 38% (2644 particles of 6930), 60% (6730 particles of 11 212), 55% (5804 particles of 10 640) and 58% (4984 particles of 8622) for each condition (no nucleotide added, ADP, ADP–AlFx and ADP–BeFx, respectively). Top views cannot be assigned to single or double-ring complexes and to compact or stretched conformations. Reference-free averages were similar in all conditions tested (Figure 4B).

### Modelling the conformational transitions of RuvBL1–RuvBL2

**Figure 5.**
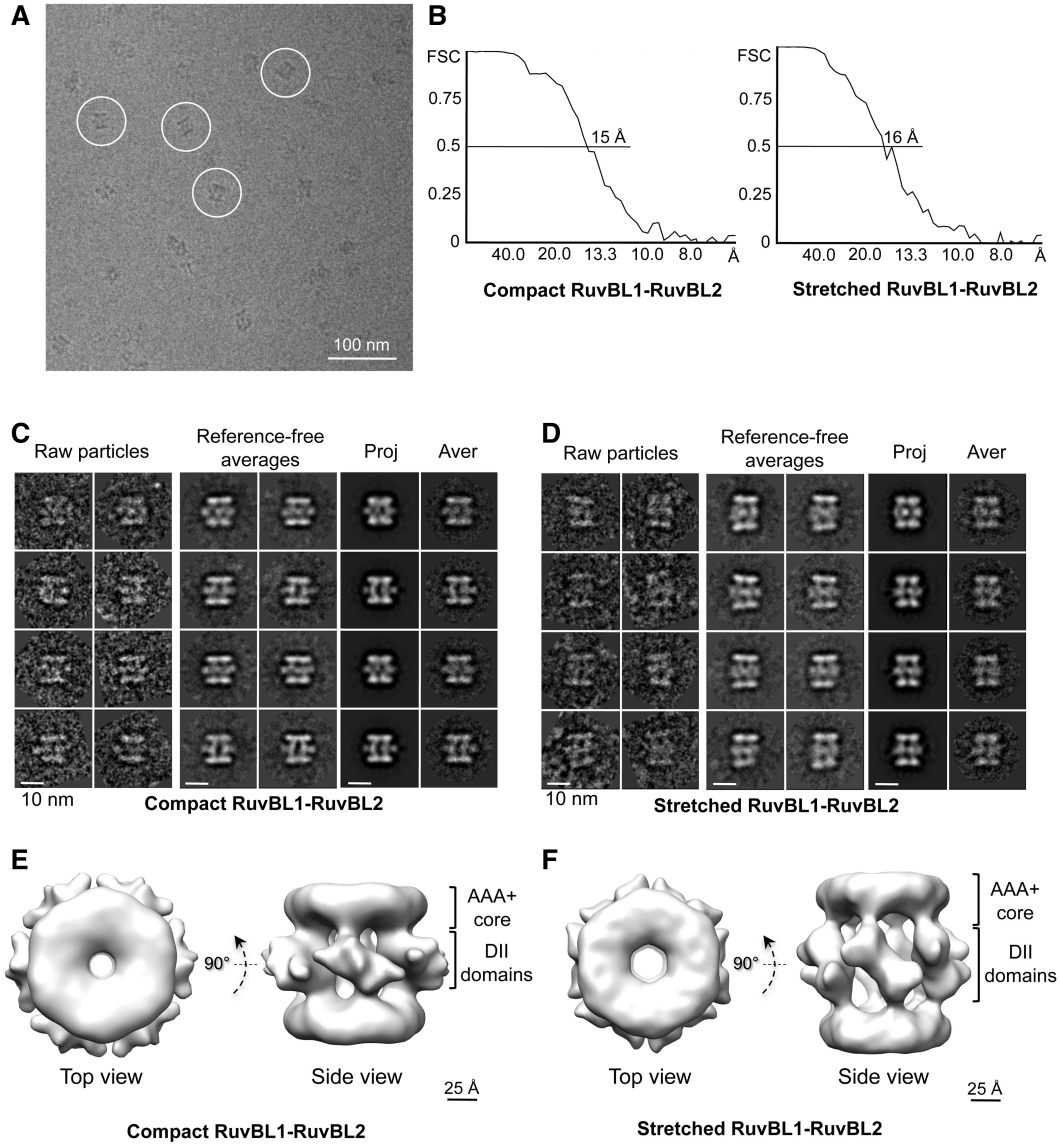
Cryo-EM structures of the human RuvBL1–RuvBL2 complex. (**A**) Typical field of frozen-hydrated RuvBL1–RuvBL2 dodecamers. Representative particles are highlighted within circles. For better visualization, we selected a micrograph taken at ∼4 μm defocus, and the protein density is shown in black. Scale bar, 100 nm. (**B**) Resolution estimate for the compact and stretched RuvBL1–RuvBL2 structures using the FSC method. (**C**) Compact conformation: raw particles, reference-free averages and pairs of projections (Proj) and averages (Aver) obtained after angular refinement. Scale bar, 10 nm. (**D**) Stretched conformation: raw particles, reference-free averages and pairs of projections (Proj) and averages (Aver) obtained after angular refinement. Scale bar, 10 nm. (**E**) Two views of the RuvBL1–RuvBL2 complex (compact conformation). Scale bar, 2.5 nm. (**F**) Two views of the RuvBL1–RuvBL2 complex (stretched conformation). Scale bar, 2.5 nm.

Images of the RuvBL1–RuvBL2 complex were also obtained from vitrified samples in the absence of staining (cryo-EM) to increase the resolution of the structures and model the structural basis of the conformational flexibility (Figure 5). A total of 24 500 images were extracted from the cryo-EM micrographs (Figure 5A), classified and averaged. Images corresponding to compact (52% of the data set) or extended (27% of the data set) side views were split and processed independently resulting in two structures at 15 Å and 16 Å, respectively (Figure 5). The vast majority of side view images could be assigned to either the extended or the compact structures, suggesting no other conformation is present in our preparation in significant amounts (Figure 5). Some side-view averages displayed a slight asymmetry between the two ends of the molecule. We determined that this was not because of a different conformation of the two rings. The refinement in 3D (see later in the text) defined these images as slightly tilted versions of a perfect side view. Top views could not be ascribed to either of these two conformations, and they were excluded from the analysis.

We evaluated the possibility of intermediate conformations between the compact and stretched structures. A maximum likelihood approach (33) and a multi-reference refinement strategy using the *multi-refine* command in EMAN (28) revealed that our data set was only represented by the compact and stretched conformations. Thus, conformational transitions between these two conformations were non-existent or not sufficiently populated to be detected with the number of particles collected.

**Figure 6.**
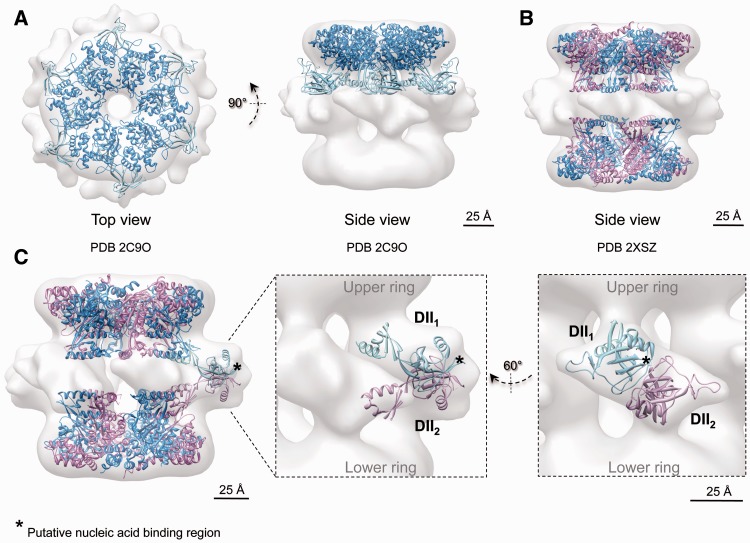
Cryo-EM structure and pseudo-atomic model of the compact conformation of human RuvBL1–RuvBL2. (**A**) Top and side views showing the fitting of the atomic structure of RuvBL1 (PDB code: 2C9O) (16) in the top ring. (**B**) Fitting of the atomic structure of the truncated human RuvBL1–RuvBL2 complex (PDB code: 2XSZ) (17). RuvBL1 is shown in blue and RuvBL2 in pink. Scale bar, 2.5 nm. (**C**) DII domains were fitted into the density of the cryo-EM map assigned to these domains after splitting external and internal regions. An asterisk points to putative regions of DII domains involved in the interaction with nucleic acids, hypothesized based on the comparison with RPA (see text). Scale bar, 2.5 nm.

**Figure 7.**
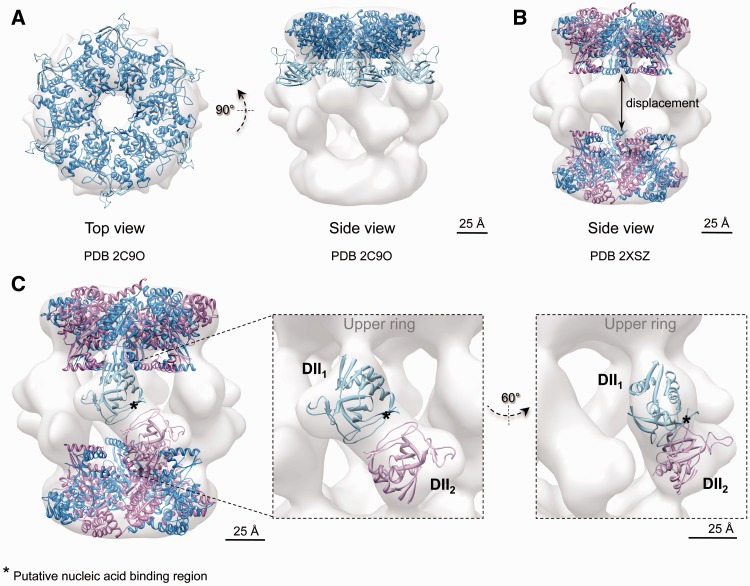
Cryo-EM structure and pseudo-atomic model of the stretched conformation of human RuvBL1–RuvBL2. (**A**) Top and side views showing the fitting of the atomic structure of RuvBL1 (PDB code: 2C9O) (16) in the top ring. (**B**) Fitting of the atomic structure of the truncated human RuvBL1–RuvBL2 complex (PDB code: 2XSZ) (17). RuvBL1 is shown in blue and RuvBL2 in pink. The two rings were moved apart to accommodate the distance between rings in the crystal structure to that observed in the stretched conformation. Scale bar, 2.5 nm. (**C**) DII domains were fitted into the density of the cryo-EM map assigned to these domains after splitting external and internal regions. An asterisk points to putative regions of DII domains involved in the interaction with nucleic acids, hypothesized based on the comparison with RPA (see text). Scale bar, 2.5 nm.

The AAA+ core showed an excellent agreement with the crystal structure of RuvBL1 (16) in the compact (cross-correlation = 0.87) (Figure 6A) and the stretched conformations (cross-correlation = 0.87) (Figure 6A); thus, they can be considered identical at this resolution. The size of the channel at the centre of the ring would permit the access of an ssDNA/ssRNA, whereas a dsDNA/dsRNA would not fit the space available (16). The overall dimensions of the truncated RuvBL1–RuvBL2 dodecamer (17) were found to match closely to those of the compact conformation (Figure 6B), whereas the two AAA+ rings were moved apart significantly to accommodate to their position in the stretched conformation (cross-correlation = 0.92 after displacing the two rings) (Figure 7B).

DII domains displayed different conformations in compact and stretched structures, which were also distinct to the conformation described in the crystal structure of the homo-hexameric RuvBL1 complex (16). We modelled these conformational changes by fitting the atomic structure of the DII domain of RuvBL1 (16) within the compact (Figure 6C) and stretched structures (Figure 7C). For this purpose, DII domains were split in half corresponding to the external and internal regions, and each region was fitted independently into the EM density. The positions of the manually placed DII domains were refined using Chimera (30) to optimize the correlation between the crystal structure and the EM density while minimizing the movement of DII compared with the crystal structure of RuvBL1 (16) and maintaining the connectivity between the external and internal regions of DII. In the compact structure, the internal regions of the DII domains from opposite subunits were found in close proximity. This conformation is compatible with the interaction between internal regions of DII domains proposed in the atomic structure of the truncated RuvBL1–RuvBL2 complex (17) (Figure 6C).

Our structural model of the RuvBL1–RuvBL2 docecamer builds on the observation in the crystal structure that each RuvBL1 subunit from one ring contacts a RuvBL2 subunit from the opposite ring (17), and suggests that the external regions of DII domains from these subunits are in intimate contact in the compact conformation. Such a model implies that DII domains are competent for protein–protein interactions, and a major driving force in the interaction between the two rings. Others (16) hypothesized that the DII domains are involved in nucleic acid binding based on the structural alignment of DII and RPA bound to a ssDNA (PDB code: 1JMC) (34). Residues 183–233 of RPA were superposed onto residues 127–233 of the DII domain from RuvBL1, and the comparison between the two structures pointed at the putative DNA/RNA binding region in DII (Figure 6C, labelled with an asterisk in RuvBL1).

Fitting experiments performed in the stretched conformation (Figure 7C) revealed that DII domains are pulled upwards and slightly rotated, driving the two AAA+ rings further apart. Such conformational transitions drive the putative nucleic acid binding regions of the DII domains from a tight contact in the compact structure (Figure 6C, labelled with an asterisk in RuvBL1) to a looser interface in the stretched structure (Figure 7C, labelled with an asterisk in RuvBL1; Supplementary Movie S1).

## DISCUSSION

RuvBL1 and RuvBL2, two AAA+ ATPases related to prokaryotic RuvB, have been implicated in essential cellular processes as diverse as DNA repair, transcription, chromatin remodelling, NMD and telomerase assembly, among others (1,3). Such functional diversity arises from their contribution to several large macromolecular complexes, including SWR1 and related chromatin remodelling complexes, association and regulation of transcription factors, such as c-myc and β-catenin, complexes in the maturation of snoRNPs and in telomerase assembly (1,3). Despite their profound biological importance, substantial controversy surrounds the structures of RuvBL1 and RuvBL2 (17,20,23,24). As a result, the molecular bases of their essential functions remain poorly understood. Together, our biochemical and structural data characterizing the human RuvLB1–RuvLB2 complex has clarified many of these outstanding issues.

There has been some discussion of the role that affinity tags used for the purification of the RuvBL1–RuvBL2 complex play in the stability of the dodecameric complex. Cheung *et al.* (23) have reported that yeast double-rings were present only when the recombinant proteins contained an N-terminal histidine tag. In contrast, the approximate 1:6:6 ratio for Arp5:Rvb1:Rvb2 estimated in yeast complexes would be consistent with one Arp5 molecule per double ring in yeast (14). The human RuvBL1–RuvBL2 double-ring complex with truncated DII domains was stable after removing the histidine tag (17). In addition, double-ring complexes have been observed for proteins tagged either at the subunit’s N-terminus (16,24) or C-terminus (19,21). N- and C-terminal ends of RuvBL1 and RuvBL2 are facing opposite sides of the hexamer (17). The C-terminal end is projecting outwards from the top of the ring (Figure 1A) precluding a role of the tag in the conformation of the DII domains. We now show that the human RuvBL1–RuvBL2 double-ring complex is maintained as a dodecameric assembly after removing the histidine tag. However, the tag seems to influence the stability of the dodecamer, as double-ring complexes are more prone to disassembly in a SEC experiment after tag removal. Taken all available evidence as a whole, double rings are one of the forms of the RuvBL1–RuvBL2 complex, at least in humans. The tag seems to influence the single- to double-ring transition when assembled *in vitro*. However, this effect is probably indirect in combination with other factors, including protein concentration and the presence of nucleotides. In addition, the relevant oligomeric state is likely dependent on the interaction with other proteins as part of larger macromolecular complexes.

The ∼15 Å structure of the human RuvBL1–RuvBL2 double-ring complex (compact conformation) is compatible with the high-resolution structures solved by X-ray crystallography (16,17). The AAA+ core is a compact ring, identical at this resolution to the crystal structure of RuvBL1 hexamers (16). The central channel shows comparable dimensions in the structures solved by cryo-EM (this work) and X-ray crystallography (16,17). It has been speculated that this channel could bind RNA/DNA, as it is the case in other AAA+ proteins; however, these dimensions would restrict binding to ssDNA or ssRNA (16). The DII domains project towards the interface between the two rings, stabilizing their interaction. The distance between the AAA+ rings at both ends of the complex is similar to that found in the truncated RuvBL1–RuvBL2 double ring (17). In addition, we now resolve the DII domains previously missing in the crystal structure. Our structural model implies that DII domains are involved in protein–protein interactions between the two rings and suggests that these domains could also interact with other proteins as part of larger assemblies.

Although consistent with X-ray structures of RuvBL1 (16) and the truncated RuvBL1–RuvBL2 complex (17), our cryo-EM reconstruction of human RuvBL1–RuvBL2 differs significantly from the previously reported negative stain EM map (19). In contrast to the structure reported by Puri *et al.*, the AAA+ ring of RuvBL1 is compact, without spacing between subunits [this study, (16)]. Furthermore, the raw data presented by Puri *et al.* cannot be rationalized based on the structure of the human complex we reconstructed. They show images that differ significantly from the typical side views we observe (19). Importantly, we do not detect a significant number of cryo-EM images that cannot be assigned to either typical top or side views. Thus, our data are not compatible with alternative structural models. A potential source of errors in their analysis could arise from the lack of perfect side views in their data set and/or a mixture of conformations. Alternatively, they could have solved a completely different conformation of the complex as suggested by Niewiarowski *et al.* (21). Nevertheless, we claim that the close agreement between our cryo-EM structure and the atomic structures for RuvBL1 and RuvBL1–RuvBL2 is a strong support for the validity of our structural analysis reported here.

In this work, rotational symmetry is assumed during refinement; thus, we cannot formally rule out that each DII domain could potentially display a different conformation. We evaluated this possibility by first applying rotational symmetry during refinement until convergence. Then, the structures were refined further without imposing any symmetry, and we found that the structure of the complex remained unchanged. This suggests that any fluctuations of the 6-fold symmetry and/or the symmetry between the two rings were not detected at the resolution of these images. A recent work modelled extensive flexibility of the DII domains within hexameric complexes (35). We predict that the conformational flexibility of DII domains within the double-ring RuvBL1–RuvBL2 complex is possibly more restricted than in the single-ring complex, as these domains are involved in inter-ring interactions.

**Figure 8.**
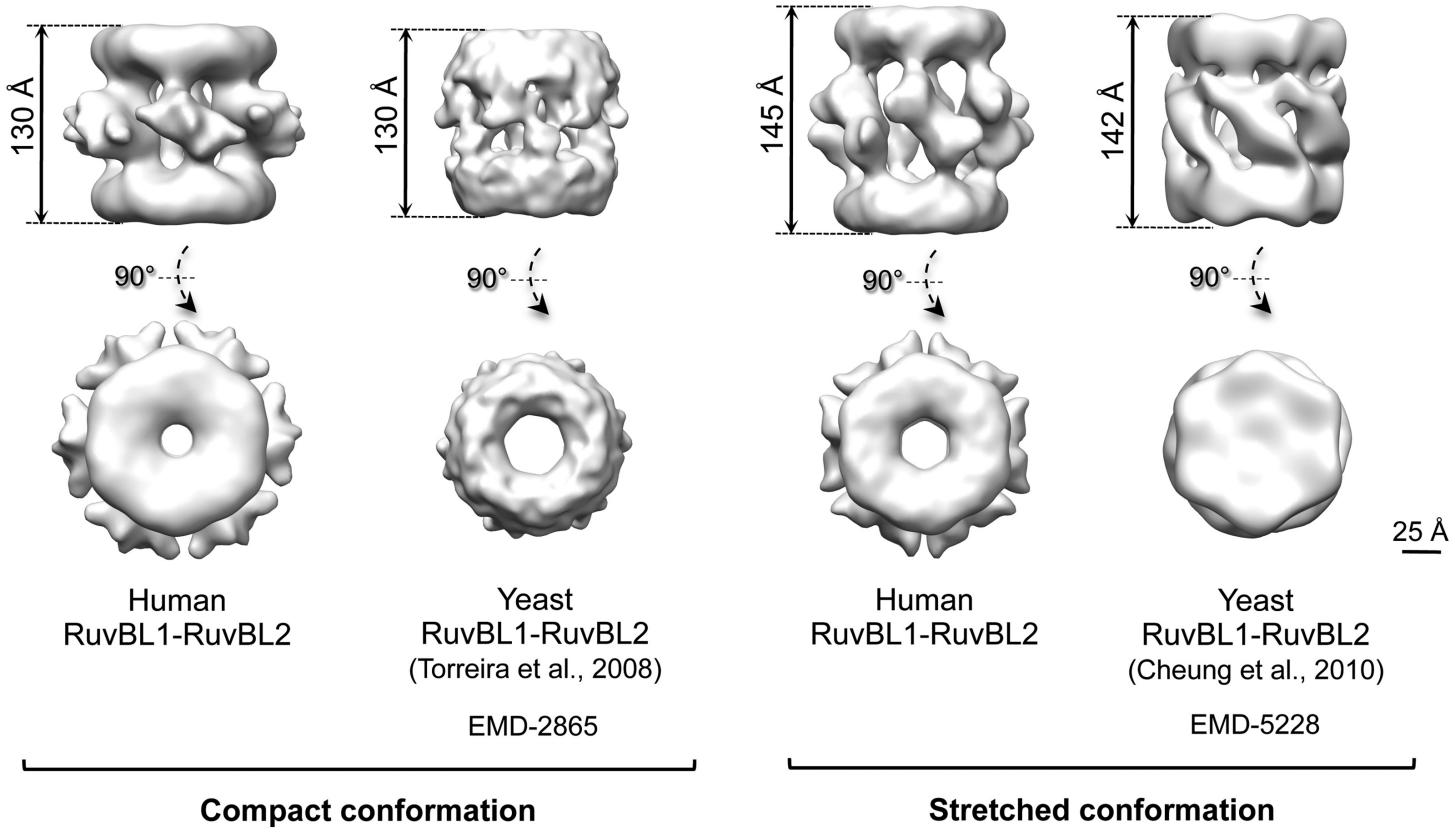
Comparison between the compact and stretched conformations of the human and yeast RuvBL1–RuvBL2 complexes solved by EM. Scale bar, 2.5 nm.

A key finding in this study is that the RuvBL1–RuvBL2 dodecamer is present in two coexisting conformations, compact and stretched. Interestingly, these conformational changes are not directly related to a certain nucleotide state in the AAA+ ring, as both conformations are found in the presence of different nucleotides. We propose that this is the major reason behind the divergent structural models previously put forth (Figure 8). In yeast, double-ring complexes have been described as either stretched (23) or compact (24), whereas here, we show that these states can coexist simultaneously in the human system, strongly suggesting that the complex alternates between the two conformations. It is likely that the different yeast structures reflect an enrichment of one or the other transition states. This raises the possibility of artifacts in those reconstructions derived from samples containing a mixture of conformations, if this heterogeneity is not taken account. In this work, we have addressed this issue by processing only homogenous subsets of particles exhaustively classified in 2D. This heterogeneity might also partly explain some of the difficulties in crystallizing the full-length complex (17).

We model the conformational changes in the DII domain accounting for these transitions (Figures 6 and 7). In the compact conformation, the external regions of the DII domains are intimately inter-connected at the centre of the molecule, which results in a significant shortening of the length of the complex. Meanwhile, the internal regions of DII are sufficiently close to account for the contacts described in the structure of the truncated RuvBL1–RuvBL2 dodecamer (17). In the stretched conformation, DII domains are tilted upwards and rotate slightly, moving the AAA+ rings apart. Concurrently, the internal regions of the DII domains are displaced, making the contact between these regions from opposite rings unlikely. We find that most of the RuvBL1–RuvBL2 images in our data set accommodate these two structures, and we do not detect intermediate conformations. This suggests that these conformations are stabilized by a certain arrangement of the DII domains, and intermediates between these extremes are less abundant. Nonetheless, we cannot discard that a wider range of conformations might be potentially possible. In fact, the compact conformation of the yeast complex described by Torreira *et al.* (24) shows a wider central opening of the AAA+ ring (Figure 6), suggesting other conformations are also plausible.

What is the functional significance of these conformational transitions? The DII domain is structurally similar to the ssDNA-binding domain in RPA. In addition, the isolated DII domain from RuvBL1 has been shown to bind ssDNA, dsDNA and ssRNA *in vitro* (16). We find that the putative regions involved in nucleic acid binding seem more exposed in the stretched conformation compared with the compact conformation. Hence, we propose that these conformational changes might regulate the interaction with nucleic acids. Alternatively, the interaction between DII domains and DNA or RNA might shift the equilibrium of the conformations to favour one state, regulating the functions of the complex. In addition, DII domains are implicated in protein–protein interactions between the two rings; thus, a more general role of these domains as protein-binding modules within larger macromolecular complexes could be possible. Each DII domain is directly connected to the ATPase core of each monomer, and it is reasonable to hypothesize that there is a link between the conformation of the AAA+ core and the DII domains. Conformational changes in DII domains could regulate the ATPase activity and/or *vice versa*. Some evidence suggests that DII domains modulate the ATPase and helicase activity of the RuvBL1–RuvBL2 complex (16,17), and this could indicate that large conformational changes within the dodecamer could have a wider impact on the functionally of the RuvBL1–RuvBL2 complex.

In this work, we reveal the structure of the full-length human RuvBL1–RuvBL2 double-ring complex, and we resolve the discrepancy for the different structures described. We show that RuvBL1–RuvBL2 displays two distinct conformations and propose that these conformational changes can have a functional impact in the context of the large complexes containing RuvBL1–RuvBL2. These conformational transitions could be part of the mechanism of ‘remodelling’ by converting the complex from one state to another, and all these changes could be somehow interconnected with a modulation of the ATPase activity, thanks to the connection between the DII domains and the AAA+ core.

## ACCESSION NUMBERS

The cryo-EM structures of the compact and stretched conformations of the RuvBL1–RuvBL2 complex have been deposited in the Electron Microscopy Data Bank (EMDB) (http://www.emdatabank.org) with accession codes EMD-2163 and EMD-2164, respectively.

## SUPPLEMENTARY DATA

Supplementary Data are available at NAR Online: Supplementary Figure 1 and Supplementary Movie 1.

## FUNDING

Spanish Government [SAF2008-00451 to O.L., SAF2011-22988 to O.L.]; [BES-2009-014133 to A.L-P.]; ‘Red Temática de Investigación Cooperativa en Cáncer (RTICC)’ [RD06/0020/1001]; Human Frontiers Science Program [RGP39/2008]; ‘Ramón Areces’ Foundation. Funding for open access charge: Spanish Government [SAF2011-22988 to O.L.].

*Conflict of interest statement*. None declared.

## Supplementary Material

Supplementary Data
