# Supplementary material for: Conformational transitions regulate the exposure of a DNA-binding domain in the RuvBL1–RuvBL2 complex
Source: Nucleic Acids Res. 2012 Sep 21;40(21):11086–99. doi: 10.1093/nar/gks871 (PMC3510503; doi:10.1093/nar/gks871)
Supplement: Supplementary Data [file supp_40_21_11086__index.html]

Conformational transitions regulate the exposure of a DNA-binding domain in the RuvBL1–RuvBL2 complex — Supplementary Data 

# Conformational transitions regulate the exposure of a DNA-binding domain in the RuvBL1–RuvBL2 complex

## Supplementary Data

files

**Files in this Data Supplement:**

- Supplementary Data - pdf file
