# Supplementary material for: Conformational transitions regulate the exposure of a DNA-binding domain in the RuvBL1–RuvBL2 complex
Source: Nucleic Acids Res. 2012 Sep 21;40(21):11086–99. doi: 10.1093/nar/gks871 (PMC3510503; doi:10.1093/nar/gks871)
Supplement: Supplementary Data [file supp_gks871_nar-01544-h-2012-File010.pdf]

SUPPLEMENTARY INFORMATION

Conformational transitions regulate the exposure of a DNA binding domain in the RuvBL1-RuvBL2 complex

Andrés López-Perrote, Hugo Muñoz-Hernández, David Gil and Oscar Llorca

Supplementary Figure 1. Electron microscopy of two conformations of the human RuvBL1-RuvBL2 complex. (**A**) The resolution of the 3D reconstructions for the compact and stretched conformations obtained after negative stain was evaluated using the Fourier Shell Correlation (FSC) method, and estimated using a 0.5 FSC cutoff as 26 Å and 30 Å respectively. (**B**) Fitting of the atomic structure of RuvBL1 (PDB 2C9O) and the truncated RuvBL1-RuvBL2 dodecamer (PDB 2XSZ) into the EM map of the compact conformation. Correlation values between the EM reconstruction and the crystal structures are indicated. Scale bar represents 2.5 nm. (**C**) Fitting of the atomic structure of the truncated RuvBL1-RuvBL2 dodecamer (PDB 2XSZ) into the EM map of the stretched conformation. The correlation value between the EM reconstruction and the crystal structure is indicated. Scale bar, 2.5 nm.

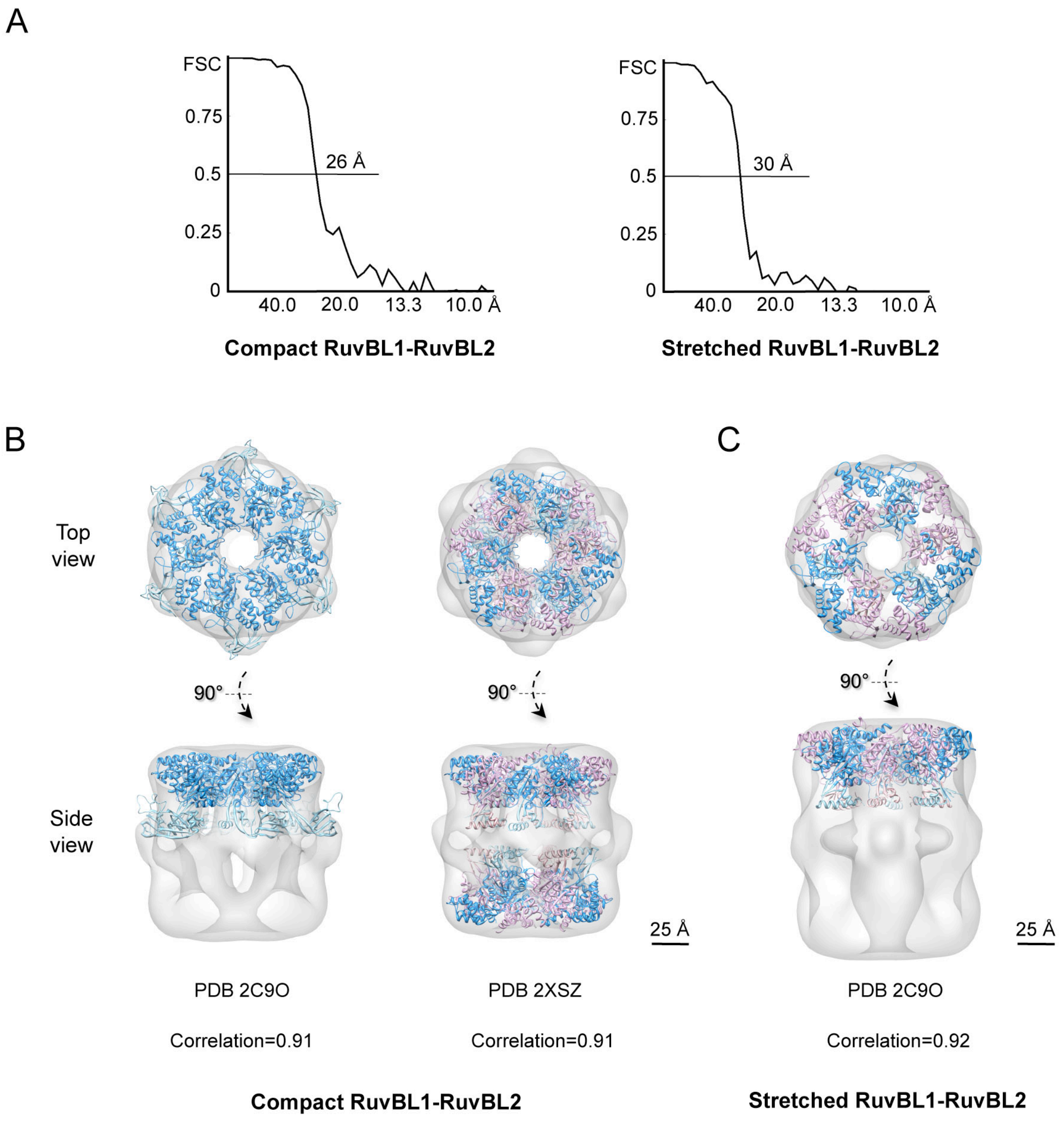
A
FSC
0.75
0.5
0.25
0
26 Å
40.0
20.0
13.3
10.0 Å
Compact RuvBL1-RuvBL2
FSC
0.75
0.5
0.25
0
30 Å
40.0
20.0
13.3
10.0 Å
Stretched RuvBL1-RuvBL2
B
C
Top view
Side view
90°
90°
90°
25 Å
25 Å
PDB 2C9O
PDB 2XSZ
PDB 2C9O
Correlation=0.91
Correlation=0.91
Correlation=0.92
Compact RuvBL1-RuvBL2
Stretched RuvBL1-RuvBL2
